# Obstetric Outcomes of Mothers Previously Exposed to Sexual Violence

**DOI:** 10.1371/journal.pone.0150726

**Published:** 2016-03-23

**Authors:** Agnes Gisladottir, Miguel Angel Luque-Fernandez, Bernard L. Harlow, Berglind Gudmundsdottir, Eyrun Jonsdottir, Ragnheidur I. Bjarnadottir, Arna Hauksdottir, Thor Aspelund, Sven Cnattingius, Unnur A. Valdimarsdottir

**Affiliations:** 1 Center of Public Health Sciences, University of Iceland, Reykjavik, Iceland; 2 London School of Hygiene and Tropical Medicine, Department of Non-Communicable Diseases Epidemiology, London, United Kingdom; 3 Harvard School of Public Health, Boston, Massachusetts, United States of America; 4 Boston University School of Public Health, Boston, Massachusetts, United States of America; 5 Rape Trauma Service and the Trauma Center, Landspitali - The National University Hospital of Iceland, Reykjavik, Iceland; 6 Psychology Department, University of Iceland, Reykjavik, Iceland; 7 Department of Obstetrics and Gynecology, Landspitali - The National University Hospital of Iceland, Reykjavik, Iceland; 8 The Icelandic Heart Association, Kopavogur, Iceland; 9 Unit of Clinical Epidemiology, Karolinska Institutet, Stockholm, Sweden; 10 Department of Medical Epidemiology, Karolinska Institutet, Stockholm, Sweden; University of Rennes-1, FRANCE

## Abstract

**Background:**

There is a scarcity of data on the association of sexual violence and women's subsequent obstetric outcomes. Our aim was to investigate whether women exposed to sexual violence as teenagers (12–19 years of age) or adults present with different obstetric outcomes than women with no record of such violence.

**Methods:**

We linked detailed prospectively collected information on women attending a Rape Trauma Service (RTS) to the Icelandic Medical Birth Registry (IBR). Women who attended the RTS in 1993–2010 and delivered (on average 5.8 years later) at least one singleton infant in Iceland through 2012 formed our exposed cohort (n = 1068). For each exposed woman's delivery, nine deliveries by women with no RTS attendance were randomly selected from the IBR (n = 9126) matched on age, parity, and year and season of delivery. Information on smoking and Body mass index (BMI) was available for a sub-sample (n = 792 exposed and n = 1416 non-exposed women). Poisson regression models were used to estimate Relative Risks (RR) with 95% confidence intervals (CI).

**Results:**

Compared with non-exposed women, exposed women presented with increased risks of maternal distress during labor and delivery (RR 1.68, 95% CI 1.01–2.79), prolonged first stage of labor (RR 1.40, 95% CI 1.03–1.88), antepartum bleeding (RR 1.95, 95% CI 1.22–3.07) and emergency instrumental delivery (RR 1.16, 95% CI 1.00–1.34). Slightly higher risks were seen for women assaulted as teenagers. Overall, we did not observe differences between the groups regarding the risk of elective cesarean section (RR 0.86, 95% CI 0.61–1.21), except for a reduced risk among those assaulted as teenagers (RR 0.56, 95% CI 0.34–0.93). Adjusting for maternal smoking and BMI in a sub-sample did not substantially affect point estimates.

**Conclusion:**

Our prospective data suggest that women with a history of sexual assault, particularly as teenagers, are at increased risks of some adverse obstetric outcomes.

## Introduction

Violence, including sexual violence, is a worldwide public health problem [1]. Exposure to sexual violence has been linked with increased risks of various negative physical [2–4] and mental [5–7] health consequences. Various mechanisms have been hypothesized, including biological, behavioral and emotional, yet further studies are needed to better understand and act on these complex pathways [8]. Associations have also been reported between exposure to sexual violence and subsequent risk factors such as illicit drug- or alcohol use [5, 9], smoking [9] and high body mass index (BMI)[10]; associations that also have been observed among women in subsequent pregnancies [11–15].

Sexual violence involves the body parts of human reproduction and indeed some survivors of rape have described re-experiencing the assault during labor and delivery [16]. Although the number of studies has increased during the last decade, knowledge on the potential influence of sexual violence on maternal and obstetric outcomes is still scarce. Women who have been exposed to sexual violence have been reported to be at increased risk of being hospitalized during pregnancy [17, 18], reporting common pregnancy complaints (for example nausea, backache, and tiredness) [12, 13] and fearing childbirth before [19] or during labor [20].

Few studies have addressed whether a history of sexual violence increases the risk of labor complications, such as labor dystocia; some studies have reported prolonged second stage of labor in women exposed in adulthood [21, 22], while others have not found any associations between sexual violence and labor dystocia [14, 23]. Similarly, findings from studies on the association between sexual violence and risk of cesarean section or instrumental vaginal deliveries are mixed. Some studies have reported an association (at least for subgroups of exposed women) with elective cesareans [14, 24], emergency cesareans [14], or either [21, 22], while others have not found such associations [15, 25]. Vaginal instrumental deliveries have in some studies been associated with a history of sexual violence [21, 22] while such an association has not been confirmed in other studies [14, 15, 24]. These contradictory findings inevitably reflect the vast methodological challenges in most previous studies, including retrospective self-reports of exposure [12–15, 18–20, 23–25] or selection of small clinical samples, limiting the generalizability of the findings [21, 22]. Furthermore, some [12, 17, 25] but not all [11, 13–15, 18–24] previous studies have been restricted to sexual abuse during childhood. A recent report from the World Health Organization calls for increased research to fill existing knowledge gap on the health consequences of sexual violence [1]. Thus, to add to previous studies on obstetric outcomes among sexual violence survivors, larger population-based follow-up studies of medically registered indices of sexual violence are needed for improved understanding, and prevention, of potential adverse consequences in pregnancy and labor.

In Reykjavik, Iceland, prospectively collected information on women seeking trauma services for sexual assault is available from 1993 and onwards, providing an opportunity for complete follow-up of subsequent births in the nationwide electronic Icelandic Medical Birth Registry (IBR). Using these resources, it is possible to conduct studies that complement some of the methodological shortcomings of many previous studies. We aimed to investigate the associations between women’s sexual violence exposure as teenagers or adults and adverse obstetric outcomes later in their lives. We further aimed to identify whether the risk of adverse outcomes varied by age at and time from sexual violence exposure.

## Materials and Methods

The study was accepted by The National Bioethics Committee (VSNb2010050009/03.7) and The Data Protection Authority (2013040591HGK/—), with acceptance from Landspitali and The Directorate of Health. No participants were contacted; the data were analyzed anonymously.

The Rape Trauma Service (RTS) opened in March 1993 at the accident and emergency department of Landspitali—the National University Hospital of Iceland. The RTS provides emergency services for sexually assaulted individuals who are 12 years old and older, of whom most report severe sexual violence (penetration). The data on assaulted women are registered by attending health care professionals independent of this study. The services and data registration are further described in our previous study [26]. Upon attendance to the RTS, individuals are registered with their unique personal identification number. For the preparation of the exposed cohort, a data administrator at the hospital performed a record linkage of the unique identification numbers of women attending RTS from 1993 to 2011 to the Icelandic Birth Register to obtain information on obstetric outcomes. In total, 670 exposed women with Icelandic citizenship delivered 1068 singleton infants in Iceland in the period following attendance until the end of follow-up (December 31^st^ 2012). Time from assault to delivery was on average 5.8 years (median 5.1 years, interquartile range 5.6 years).

We considered all mothers in the IBR who had not attended the RTS through Dec 31^st^ 2011 as being non-exposed. For each exposed mother’s delivery, we aimed to randomly select ten deliveries, by Icelandic mothers, with a computerized program for our non-exposed cohort, with the following matching criteria: *Age* (a) ≤19 years, b) 20–39 years, matched on exact age, c) ≥40 years), *parity* (primipara or multipara), and *calendar season of delivery* (January-April, May-August, or September-December of the same year). We restricted our analysis to singletons and, if randomly selected more than once, applied each delivery by a non-exposed mother only once in our analyses. This resulted in a study population of 9126 deliveries of non-exposed mothers and 1068 deliveries of exposed mothers.

The IBR includes information on every delivery in Iceland from gestational week 22. Data is registered by health professionals at each antenatal clinic and birthplace, and is sent electronically after delivery to the IBR, where it is verified by a secretary. Variables include information on mother and infant, International Classification of Diseases codes for all diagnosis (ICD) and NOMESCO Classification of Surgical Procedures (NCSP) codes. All earlier ICD codes in the IBR have been updated according to the tenth version (ICD -10).

ICD—10 codes were used to define the following outcomes: *Labor dystocia* (primary (O62.0), secondary (O62.1) and unspecified (O62.2) dystocia, prolonged first (O63.0) and second (O63.1) latent phase, or unspecified prolonged labor (O63.9)), *maternal distress during labor and delivery* (O75.0), *antepartum bleeding* (O44-46, including placental abruption O45), *elective caesarean section* (O82.0), and *emergency caesarean section* (O82.1).

The following NCSP codes were used to define obstetric interventions: *Induced labor* (MAXC00, 02, 09), *instrumental vaginal delivery* (either forceps or vacuum extraction) (MASE00, 03, 96; MASF00, 10, 96, MASG03, 13, or ICD-10 O81.0, O81.1, O81.2, O81.3, O81.4, O81.5), and *emergency instrumental delivery* (defined as either vaginal instrumental delivery or emergency cesarean delivery).

Women’s occupation (registered in the IBR) was roughly categorized for this study as being a student, not working (unemployed, full-time at home or on disability benefit) and employed/missing (missing reported in our previous article <2–4%).

### Statistical analysis

Using Poisson log-linear models, we assessed differences in risk of labor characteristics and delivery interventions between exposed and non-exposed cohorts. Results are presented as prevalence rate ratios or relative risks (RR) with 95% confidence intervals (CI). Due to the matched design, we inherently adjusted for age, parity, and season of delivery in our crude models. In our secondary models, we additionally adjusted for socio-economic factors (marital and occupational status). We further analyzed the data stratified on: a) Woman’s age at the time of attendance to the RTS; *teenagers* (12–19 years of age, median 17years) or *adults* (≥20 years, median 23 years), which reflects before and after the general age at which most Icelandic students complete a four-year elective high-school education. b) Time from exposure to delivery; ≤5 or >5 years, which reflects the median time from assault to delivery, 5.1 years. The time stratified analysis were adjusted for maternal age, parity (nulliparous or multiparous), and year of delivery. Women who had an elective cesarean section were excluded from other analyses.

#### Additional analysis

The IBR does not include electronic information about smoking and BMI. However, we have information about these manually collected variables in our previous study about risk factors and health during pregnancy [11]. This previous study consisted of a sub-sample of exposed women within our current dataset, those who attended the RTS through 2008 and delivered to mid-April 2011, while the non-exposed women differ from those in the current study (due to different matching criteria and fewer non-exposed women in the previous study). The results of the previous study [11] showed that exposed women were at increased risk of sustained smoking during pregnancy as well as marginally higher risk of obesity when compared to non-exposed women. Therefore, to explore the role of these risk factors for labor and delivery outcomes, we performed additional analyses and show RRs with and without adjustment for smoking and BMI in the sub-sample (n = 792 exposed and n = 1416 non-exposed women).

Data analysis was conducted with IBM SPSS Statistics version 20.0 (Armonk, NY: IBM Corp.).

## Results

Table 1 shows the matching variables and other background characteristics between exposed and non-exposed women. The three matching variables, maternal age, parity, and season, were comparable between the exposed and non-exposed cohorts. Compared to non-exposed women, exposed women were less frequently employed or cohabiting, and a higher proportion of their deliveries occurred in the capital.

**Table 1:** Background characteristics comparing women exposed versus non-exposed to sexual violence.

	Non-exposed		Exposed	
	n	%	n	%
	9126	89.5%	1068	10.5%
**Age at delivery (years)**				
≤19	955	10.5%	119	11.1%
20–24	3085	33.8%	368	34.5%
25–29	2906	31.8%	335	31.4%
30–34	1571	17.2%	176	16.5%
≥35	609	6.7%	70	6.6%
**Parity**				
Primiparas	4308	47.2%	504	47.2%
Multiparas	4818	52.8%	564	52.8%
**Season of delivery**				
January–April	2865	31.4%	343	32.1%
May–August	3126	34.3%	361	33.8%
September–December	3135	34.4%	364	34.1%
**Occupational Status**				
Employed/missing	6166	67.6%	581	54.4%
Student	2023	22.2%	228	21.3%
Not working (disability, unemployed, full time at home)	937	10.3%	259	24.3%
**Marital status**				
Cohabiting	7055	77.3%	631	59.1%
Not cohabiting	1944	21.3%	420	39.3%
Missing	127	1.4%	17	1.6%
**Place of delivery**				
Capital (Landspitali)	6123	67.1%	828	77.5%
Other	3003	32.9%	240	22.5%

Table 2 shows the prevalence and risks of the studied labor characteristics in exposed versus non-exposed women, and also in our additional analysis where information on smoking and BMI was available. Compared to non-exposed women, exposed women were at a 40 percent increased risk of having a prolonged first stage of labor, whereas no difference was found regarding prolonged second stage. Exposed women were also at 60 percent increased risk of being diagnosed with maternal distress during labor and delivery, and at a twofold risk of antepartum bleeding. We found no differences between the groups with respect to induced labor or dystocia. Adding socio-economic factors (occupation and marital status) to the models resulted in minimal change of the reported point estimates (S1 Table). Analysis restricted to the sub-sample revealed no substantial differences in point estimates when adding smoking and BMI to the model.

**Table 2:** Comparison of labor characteristics among women exposed versus non-exposed to sexual violence.

Full dataset							Sub-sample^a^							
	Non-exposed women		Exposed women				Non-exposed women		Exposed women					
	n	%	n	%	RR^b^	95% CI	n	%	n	%	RR^c^	95% CI	Adj RR^c^^d^	95% CI
**Total**^e^	**8699**	**89.5**	**1025**	**10.5**			**1530**	**63.7**	**873**	**36.3**				
**Induced labor**	2273	26.1	291	28.4	1.09	0.98–1.21	368	24.1	229	26.2	1.09	0.95–1.25	1.08	0.93–1.26
**Labor dystocia**	729	8.4	101	9.5	1.18	0.96–1.47	124	8.1	87	10.0	1.17	0.88–1.57	1.26	0.89–1.77
Prolonged first stage of labor	292	3.4	48	4.7	1.40	1.03–1.88	45	2.9	39	4.5	1.50	0.96–2.36	1.38	0.84–2.25
Prolonged second stage of labor	356	4.1	45	4.4	1.07	0.77–1.49	56	3.7	39	4.5	1.22	0.79–1.90	1.40	0.89–2.23
**Maternal distress during labor and delivery**	96	1.1	19	1.9	1.68	1.01–2.79	21	1.4	16	1.8	1.56	0.81–3.01	1.40	0.67–2.92
**Antepartum bleeding**	96	1.1	22	2.1	1.95	1.23–3.07	35	2.3	17	1.9	1.08	0.59–1.98	0.81	0.41–1.58
Placental abruption	36	0.4	7	0.7	1.66	0.74–3.72	6	0.4	6	0.7	2.38	0.69–8.26	1.72	0.45–6.53

^a^Exposed and non-exposed women within our previous dataset with manually retrieved information on smoking and BMI.

^b^Relative Risks with non-exposed women as a reference group. Data matched on age, parity and season of delivery.

^**c**^Relative Risks with non-exposed women as a reference group. Data adjusted for age, parity, year and month of delivery.

^**d**^Additionally adjusted for smoking (in 2 categories, no/quit vs. yes) and body mass index (BMI).

^e^Women who underwent elective cesarean section were excluded from all analysis in this table.

Table 3 shows the same outcomes as Table 2, now stratified by age at attendance to the RTS. Compared to non-exposed women, the relative risks were generally higher for women exposed as teenagers than for women exposed later in life; a 28% increased risk of induced labor, a 29% increased risk of labor dystocia, a 60% increased risk of having a prolonged first stage of labor, and a twofold risk of maternal distress during labor and delivery. However, the risk of antepartum bleeding was twofold increased among those assaulted in adulthood, while the risk was not significant for those exposed earlier. Among women exposed as teenagers (n = 629), 273 deliveries occurred ≤5 years of the assault and 356 occurred later. The corresponding figures for deliveries among women exposed as adults (n = 396) were 235 and 161, respectively. Time stratified analysis showed mostly comparable estimates, irrespective of the proximity from the assault to the delivery (S2 Table).

**Table 3:** Comparison of labor characteristics among women exposed versus non-exposed to sexual violence: Stratified by age at rape trauma consultation.

	Non-exposed women		Age at rape trauma consultation <20 years^a^				Age at rape trauma consultation ≥20 years^a^			
					Model				Model	
	n	%	n	%	RR^b^	95% CI	n	%	RR^b^	95% CI
**Total**	**8699**	**89.5**	**629**	**6.7**			**396**	**4.1**		
**Induced labor**	2273	26.1	192	30.5	1.17	1.03–1.33	99	25.0	0.96	0.80–1.15
**Labor dystocia**	729	8.4	68	10.8	1.29	1.01–1.65	33	8.3	0.99	0.67–1.46
Prolonged first stage of labor	292	3.4	34	5.4	1.61	1.14–2.28	14	3.5	1.05	0.62–1.79
Prolonged second stage of labor	356	4.1	26	4.1	1.01	0.68–1.51	19	4.8	1.17	0.69–1.99
**Maternal distress during labor and delivery**	96	1.1	14	2.2	2.02	1.12–3.64	5	1.3	1.14	0.47–2.78
**Antepartum bleeding**	96	1.1	12	1.9	1.73	0.96–3.12	10	2.5	2.28	1.21–4.33
Placental abruption	36	0.4	3	0.5	1.15	0.36–3.73	4	1.0	2.44	0.88–6.78

^a^Those who attended the Rape Trauma Service more than once were categorized according to age at first attendance.

^b^Relative Risks with non-exposed women as a reference group.

Table 4 shows risks of delivery interventions in women exposed to sexual violence versus non-exposed women, both in our full cohort and our sub-sample. Exposed women were at an increased risk of having emergency instrumental deliveries, compared to non-exposed women. We found no difference regarding elective cesarean sections. Additional adjustment for socio-economic status did not change the point estimates substantially (S1 Table). The point estimates were comparable in crude models and when adjusting for smoking and BMI in our sub-sample.

**Table 4:** Comparison of delivery interventions among women exposed versus non-exposed to sexual violence.

Full dataset							Sub-sample^a^							
	Non-exposed women		Exposed women				Non-exposed women		Exposed women					
	n	%	n	%	RR^b^	95% CI	n	%	n	%	RR^c^	95% CI	Adj RR^c^^d^	95% CI
**Total**	**9126**	**89.5**	**1068**	**10.5**			**1614**	**64.1**	**903**	**35.9**				
**Elective cesarean section**	427	4.7	43	4.0	0.86	0.61–1.21	84	5.2	30	3.3	0.92	0.59–1.45	1.18	0.74–1.88
**Total**^e^	**8699**	**89.5**	**1025**	**10.5**			**1530**	**63.7**	**873**	**36.3**				
**Emergency cesarean section**	842	9.7	117	11.4	1.18	0.97–1.44	161	10.5	102	11.7	1.29	0.99–1.67	1.26	0.94–1.69
**Instrumental vaginal delivery**	698	8.0	93	9.1	1.13	0.91–1.40	114	7.5	84	9.6	1.29	0.98–1.70	1.30	0.96–1.74
**Emergency instrumental delivery**^f^	1540	17.7	210	20.5	1.16	1.00–1.34	275	18.0	186	21.3	1.29	1.07–1.56	1.28	1.05–1.58

^a^Exposed and non-exposed women within our previous dataset with manually retrieved information on smoking and BMI.

^b^Relative Risks with non-exposed women as a reference group. Data matched on age, parity and season of delivery.

^c^Relative Risks with non-exposed women as a reference group. Data adjusted for age, parity, year and month of delivery.

^d^Additionally adjusted for smoking (in 2 categories, no/quit vs. yes) and body mass index (BMI).

^e^Women who underwent elective cesarean section were excluded from other analysis in this table.

^f^Either emergency cesarean section or instrumental vaginal delivery.

Table 5 shows delivery interventions according to exposure status stratified by age at attendance to the RTS. Women exposed as teenagers were less likely to have an elective cesarean section but at a 20% increased risk of emergency instrumental deliveries, compared to non-exposed women. No statistically significant differences were found when comparing women exposed in adulthood to non-exposed women. The results remained mostly constant across categories of time from assault to delivery (S3 Table).

**Table 5:** Comparison of delivery interventions among women exposed versus non-exposed to sexual violence: Stratified by age at rape trauma consultation.

	Non-exposed women		Age at rape trauma consultation <20 years^a^				Age at rape trauma consultation ≥20 years^a^			
					Model				Model	
	n	%	n	%	RR^b^	95% CI	n	%	RR^b^	95% CI
**Total**	**9126**	**89.5**	**646**	**6.3**			**422**	**4.1**		
**Elective cesarean section**	427	4.7	17	2.6	0.56	0.34–0.93	26	6.2	1.32	0.84–2.06
**Total**^c^	**8699**	**89.5**	**629**	**6.5**			**396**	**4.1**		
**Emergency cesarean section**	842	9.7	70	11.1	1.15	0.89–1.49	47	11.9	1.23	0.91–1.65
**Instrumental vaginal delivery**	698	8.0	65	10.3	1.29	1.00–1.66	28	7.1	0.88	0.61–1.28
**Emergency instrumental delivery**^d^	1540	17.7	135	21.5	1.21	1.02–1.44	75	18.9	1.07	0.85–1.36

^a^Those who attended the Rape Trauma Service more than once were categorized according to age at first attendance.

^b^Relative Risks with non-exposed women as a reference group.

^c^Women who underwent elective cesarean section were excluded from other analysis.

^d^Either emergency cesarean section or instrumental vaginal delivery.

Lastly, restricting deliveries of exposed women to each woman’s first subsequent delivery following the exposure (whether it was her first delivery or not) showed similar results overall, with somewhat less statistical power (S4 Table).

## Discussion

The findings from this register-based study indicate an increased risk of some obstetric and delivery complications among women with history of sexual violence. Exposed women, especially those assaulted as teenagers (12–19 years of age), were at increased risks of labor dystocia (mainly prolonged first stage of labor), maternal distress during labor and delivery, and emergency instrumental delivery (vaginal instrumental delivery or emergency cesarean section), compared to non-exposed women. We found no difference regarding elective cesarean section overall. However, those assaulted as teenagers were at a lower risk of elective cesareans compared to non-exposed women. Finally, exposed women (especially those assaulted 20 years or older) were at increased risk of antepartum bleeding.

Previous studies have shown that women exposed to sexual violence are at an increased risk of fear of childbirth [19, 20], which may partly explain some of our findings, i.e. with respect to longer duration of delivery [27], assisted delivery (marginally significantly) [19], and emergency cesarean section [28]. Psychological interventions for fear of childbirth have proven effective [29], and continuous one-to-one support during labor is associated with a reduced risk of instrumental deliveries and shorter labor [30]. We are not aware of other studies reporting an association between sexual assault and maternal distress during labor and delivery, nor a prolonged first stage of labor. The first stage is characterized by dilation of the cervix and includes (frequent) examinations of progress. In a qualitative study from Norway [16], rape survivors described progress examinations as an invasion and that their rape was re-activated during childbirth. They also reported feelings of helplessness and struggle, and some felt that their body refused to give birth. In addition, women exposed to sexual violence may have an impaired confidence in health care professionals [31], which may also partially explain these findings. It is therefore important that health care providers acknowledge that conducting routine clinical work without preparing the woman can have negative effects and re-activate rape experiences [16]. Stress hormones peak during labor [32] but studies are needed to examine whether possible biologic dysregulation of the stress-response systems [8] among sexually assaulted women affect the production of labor hormones, and, therefore, obstruct the progress of labor. Obesity has been associated both with sexual violence [10] and a prolonged first stage of labor [33], but our risk estimates remained similar after adjustment for BMI and smoking in the sub-sample. We found that women exposed as teenagers were at increased risk of induction of labor. This finding aligns with the results of a previous study where the authors suggested that induction of labor may act as a way to retain control [14].

Antepartum bleeding was more common among assaulted women, especially those who attended the RTS as adults. Increased risk of hospitalization due to antepartum bleeding has been reported for women with a history of sexual violence, in a dose-response manner by violence severity; adjustment for physical violence attenuated the association [18]. Placental abruption is associated with smoking, which could have attributed to some of the risk, since our former study indicates that this age group of exposed women had a high risk of persistent smoking (and illicit drug use) during pregnancy [11]. We have no information on smoking status at the time of the exposure, thus we cannot disentangle whether the women had started to smoke before or after the sexual assault.

Our findings on delivery interventions add to previous mixed findings [14, 15, 19, 24, 25]. Notably, our data suggest that women assaulted as teenagers are less likely to have an elective cesarean section, compared to non-exposed women. In contrast, two of the largest and most recent studies in this field show increased risks of elective cesarean sections, both for all exposed women [14] and for subgroups [24]. An elective cesarean section is less likely than instrumental vaginal delivery and emergency cesarean section to result in a negative birth experience [34], which are interventions that were more prevalent among our teenage-exposed women than non-exposed women. Further studies are needed to assess which obstetric interventions are most beneficial in terms of obstetric and perinatal outcomes for exposed women. Yet, our risk estimate for emergency instrumental deliveries was modest (overall RR 1.16). To avoid these interventions and the risk of negative consequences, an increased prenatal support for pregnant exposed women may be needed.

Our data suggest that age at time of sexual assault may be a critical factor for subsequent delivery outcomes. Adversities in childhood have been associated with various negative health outcomes in a dose-response manner [35]. In a recent systematic review, consistent positive associations were found between studies on childhood exposure to violence and cardio-vascular disease, whereas studies on adult exposure revealed mixed findings [36]. Also, the risk of post-traumatic stress disorder (PTSD) has been reported somewhat greater for women exposed to sexual violence in childhood than for those exposed 18 years or older [37]. Among women exposed to child sexual abuse, re-victimization in adulthood increases the association with psychiatric disorders [5]. Pathways by which women assaulted as teenagers are at greater risks for later negative health outcomes than those assaulted later are not clear, but exposure during a more sensitive age period and risk of re-victimization may contribute to the differences somewhat. Interestingly, time passed from sexual assault did not seem to affect the age-stratified analyses. The vast majority of our study population was exposed to severe sexual violence [26] which may partly explain discrepancies with previous studies.

A major strength of our study is the population-based, prospective and independent registration of exposure and outcomes by health care professionals. Using registry data, we did not have to rely on women’s participation (which can induce selection bias) or deal with recall bias from retrospective self-reports. We are unaware of other studies that have been able to utilize such data sets and methods within this field. Nevertheless, our study also has several limitations. First, some women in the non-exposed cohort may still have been exposed to severe sexual violence, yet not attended the RTS (suggested prevalence of rape/rape attempt among women after the age of 15 in the general population is 13% [38]). If there is indeed a true association between sexual violence and the tested outcomes, this would result in an underestimation of the relative risks. Second, our exposed women all disclosed their experience and sought medical attention, the vast majority within a month after the assault [26]. Some of them utilized the psychological support/ trauma focused treatment offered at the RTS. Therefore, they may have worked through the assault to a greater extent than women who had been sexually assaulted but did not attend the RTS and were thus assigned a non-exposed status in our study. This could also lead to an underestimation of the relative risks. Third, it is indeed a limitation that smoking and BMI are not electronically registered in the IBR. However, we could analyze the potential effects of these variables on our reported associations in our previous dataset where we collected and registered these variables manually [11]. It did not affect our interpretation. Finally, while our exposed cohort is well-defined, it is small, and we indeed lack statistical power to detect differences between groups for rare outcomes.

In conclusion, our findings indicate that women who were sexually assaulted as teenagers or in adulthood are, on average 5.8 years after the exposure, at increased risks of pregnancy and delivery complications such as antepartum bleeding, maternal distress, prolonged first stage of labor and emergency instrumental deliveries. Overall, women who were assaulted as teens show greater risks of adverse outcomes. Yet, the risk estimates of delivery complications were modest and most exposed women gave birth without complications. Nevertheless, further research is needed to address how these survivors can be supported during pregnancy and delivery.

## Supporting Information

S1 Table Supplementary Table A. Comparison of labor characteristics and delivery interventions among women exposed versus non-exposed to sexual violence: With adjustment for socio-economic status.
(DOCX)

S2 Table Supplementary Table B. Comparison of labor characteristics among women exposed versus non-exposed to sexual violence: By time between assault and delivery.
(DOCX)

S3 Table Supplementary Table C. Comparison of delivery interventions among women exposed versus non-exposed to sexual violence: By time from assault to delivery.
(DOCX)

S4 Table Supplementary table D. Labor characteristics and delivery interventions among women exposed versus non-exposed to sexual violence: Restricted to each exposed woman‘s first subsequent delivery following the exposure.
(DOCX)
