# Supplementary material for: Obstetric Outcomes of Mothers Previously Exposed to Sexual Violence
Source: PLoS One. 2016 Mar 23;11(3):e0150726. doi: 10.1371/journal.pone.0150726 (PMC4805168; doi:10.1371/journal.pone.0150726)
Supplement: S1 Table — Comparison of labor characteristics and delivery interventions among women exposed versus non-exposed to sexual violence: With adjustment for socio-economic status. (DOCX) [file pone.0150726.s001.docx]

| **S1. Supplementary Table A.**  **Comparison of labor characteristics and delivery interventions among women exposed versus non-exposed to sexual violence:**  **With adjustment for socio-economic status** | | | | | | | | | |
| --- | --- | --- | --- | --- | --- | --- | --- | --- | --- |
| **Non-exposed women** | | | | **Exposed women** | | | | | |
|  | **n** | **%** | **n** | | **%** | **RR^a^** | **95% CI** | **RR^ab^** | **95% CI** |
| **Total^c^** | **8699** | **89.5** | **1025** | | **10.5** |  |  |  |  |
| **Induced labor** | 2273 | 26.1 | 291 | | 28.4 | 1.09 | 0.98-1.21 | 1.10 | 0.99-1.23 |
| **Labor dystocia** | 729 | 8.4 | 101 | | 9.5 | 1.18 | 0.96-1.47 | 1.19 | 0.96-1.48 |
| Prolonged first stage  of labor | 292 | 3.4 | 48 | | 4.7 | 1.40 | 1.03-1.88 | 1.38 | 1.02-1.88 |
| Prolonged second stage  of labor | 356 | 4.1 | 45 | | 4.4 | 1.07 | 0.77-1.49 | 1.12 | 0.81-1.56 |
| **Maternal distress during**  **labor and delivery** | 96 | 1.1 | 19 | | 1.9 | 1.68 | 1.01-2.79 | 1.68 | 1.02-2.77 |
| **Antepartum bleeding** | 96 | 1.1 | 22 | | 2.1 | 1.95 | 1.23-3.07 | 1.89 | 1.17-3.07 |
| Placental abruption | 36 | 0.4 | 7 | | 0.7 | 1.66 | 0.74-3.72 | 1.57 | 0.70-3.56 |
| **Emergency cesarean section** | 842 | 9.7 | 117 | | 11.4 | 1.18 | 0.97-1.44 | 1.19 | 0.97-1.46 |
| **Instrumental vaginal delivery** | 698 | 8.0 | 93 | | 9.1 | 1.13 | 0.91-1.40 | 1.16 | 0.94-1.44 |
| **Emergency instrumental delivery^d^** | 1540 | 17.7 | 210 | | 20.5 | 1.16 | 1.00-1.34 | 1.17 | 1.02-1.36 |
| **Total** | **9126** | **89.5** | **1068** | | **10.5** |  |  |  |  |
| **Elective cesarean section** | 427 | 4.7 | 43 | | 4.0 | 0.86 | 0.61-1.21 | 0.90 | 0.63-1.28 |

^a^Relative Risks with non-exposed women as a reference group. Data matched on age, parity and season of delivery.

^b^Additionally adjusted for socio-economic status (marital status and occupation).

^c^Women who underwent elective cesarean section were excluded from all other analyses in this table.

^d^Either emergency cesarean section or vaginal instrumental delivery
