# Supplementary material for: Obstetric Outcomes of Mothers Previously Exposed to Sexual Violence
Source: PLoS One. 2016 Mar 23;11(3):e0150726. doi: 10.1371/journal.pone.0150726 (PMC4805168; doi:10.1371/journal.pone.0150726)
Supplement: S2 Table — Comparison of labor characteristics among women exposed versus non-exposed to sexual violence: By time between assault and delivery. (DOCX) [file pone.0150726.s002.docx]

**S2. Supplementary Table B.**

**Comparison of labor characteristics among women exposed versus non-exposed to sexual violence:**

**By time between assault and delivery**

|  | **Non-exposed women** | |  | **Women exposed**  **<20 years of age^a^** | | | |  | | **Women exposed**  **≥ 20 years of age^a^** | | | | | | | |  |
| --- | --- | --- | --- | --- | --- | --- | --- | --- | --- | --- | --- | --- | --- | --- | --- | --- | --- | --- |
|  |  |  |  |  |  | **Model** | |  | |  | |  | | **Model** | | | |  |
|  | **n** | **%** |  | **n** | **%** | **RR^b^** | **95% CI** |  | | **n** | | **%** | | **RR^b^** | | **95% CI** | |  |
| **Total^c^** | **8699** | **89.5** |  | **629** | **6.7** |  |  |  | | **396** | | **4.1** | |  | |  | |  |
| ***Delivery ≤5 years of the assault*** |  |  |  | **273** | **3.0** |  |  |  | | **235** | | **2.6** | |  | |  | |  |
| ***Delivery >5 years of the assault*** |  |  |  | **356** | **3.9** |  |  |  | | **161** | | **1.7** | |  | |  | |  |
| **Induced labor** | 2273 | 26.1 |  | 192 | 30.5 | 1.17 | 1.03-1.33 |  | | 99 | | 25.0 | | 0.96 | | 0.80-1.15 | |  |
| *Delivery ≤5 years of the assault* |  |  |  | 82 | 30.0 | 1.28 | 1.07-1.55 | |  | | 53 | | 22.6 | | 0.96 | | 0.77-1.20 | |
| *Delivery >5 years of the assault* |  |  |  | 110 | 30.9 | 1.06 | 0.91-1.24 | |  | | 46 | | 28.6 | | 0.99 | | 0.79-1.24 | |
| **Prolonged first stage**  **of labor** | 292 | 3.4 |  | 34 | 5.4 | 1.61 | 1.14-2.28 | |  | | 14 | | 3.5 | | 1.05 | | 0.62-1.79 | |
| *Delivery ≤5 years of the assault* |  |  |  | 14 | 5.1 | 1.39 | 0.82-2.37 | |  | | 7 | | 3.0 | | 0.92 | | 0.44-1.91 | |
| *Delivery >5 years of the assault* |  |  |  | 20 | 5.6 | 1.80 | 1.14-2.83 | |  | | 7 | | 4.3 | | 1.34 | | 0.65-2.80 | |

| **Prolonged second stage**  **of labor** | 356 | 4.1 | 26 | 4.1 | 1.01 | 0.68-1.51 | 19 | 4.8 | 1.17 | 0.69-1.99 |
| --- | --- | --- | --- | --- | --- | --- | --- | --- | --- | --- |
| *Delivery ≤5 years of the assault* |  |  | 14 | 5.1 | 0.96 | 0.57-1.62 | 7 | 3.0 | 0.73 | 0.32-1.67 |
| *Delivery >5 years of the assault* |  |  | 12 | 3.4 | 1.02 | 0.56-1.84 | 12 | 7.5 | 2.20 | 1.21-3.99 |
| **Maternal distress during**  **labor and delivery** | 96 | 1.1 | 14 | 2.2 | 2.02 | 1.12-3.64 | 5 | 1.3 | 1.14 | 0.47-2.78 |
| *Delivery ≤5 years of the assault* |  |  | 3 | 1.1 | 0.77 | 0.24-2.44 | 3 | 1.3 | 1.31 | 0.41-4.16 |
| *Delivery >5 years of the assault* |  |  | 11 | 3.1 | 2.95 | 1.54-5.66 | 2 | 1.2 | 1.43 | 0.35-5.90 |
| **Antepartum bleeding** | 96 | 1.1 | 12 | 1.9 | 1.73 | 0.96-3.12 | 10 | 2.5 | 2.28 | 1.21-4.33 |
| *Delivery ≤5 years of the assault* |  |  | 3 | 1.1 | 1.27 | 0.40-4.07 | 6 | 2.6 | 2.16 | 0.95-4.88 |
| *Delivery >5 years of the assault* |  |  | 9 | 2.5 | 2.43 | 1.23-4.80 | 4 | 2.5 | 1.73 | 0.63-4.79 |

| ^a^Those who attended more than once were categorized according to age at first attendance and date of the latest assault leading to attendance to the Rape Trauma Service. |
| --- |
| ^b^Relative Risks with non-exposed women as a reference group. Data matched on age, parity and season and year of delivery. Time stratified analyses were adjusted for age, parity and year of delivery. |
| ^c^Women who underwent elective cesarean section were excluded from all analyses in this table. |
