# Supplementary material for: Obstetric Outcomes of Mothers Previously Exposed to Sexual Violence
Source: PLoS One. 2016 Mar 23;11(3):e0150726. doi: 10.1371/journal.pone.0150726 (PMC4805168; doi:10.1371/journal.pone.0150726)
Supplement: S3 Table — Comparison of delivery interventions among women exposed versus non-exposed to sexual violence: By time from assault to delivery. (DOCX) [file pone.0150726.s003.docx]

**S3. Supplementary Table C.**

|  | **Non-exposed women** | |  | **Women exposed**  **<20 years of age^a^** | | | |  | **Women exposed**  **≥ 20 years of age^a^** | | | | | | | | |  |
| --- | --- | --- | --- | --- | --- | --- | --- | --- | --- | --- | --- | --- | --- | --- | --- | --- | --- | --- |
|  |  |  |  |  |  | **Model** | |  |  | |  | | **Model** | | | | |  |
|  | **n** | **%** |  | **n** | **%** | **RR^b^** | **95% CI** |  | **n** | | **%** | | **RR^b^** | | **95% CI** | | |  |
| **Total** | **9126** | **89.5** |  | **646** | **6.3** |  |  |  | **422** | **4.1** | |  | |  | | |  |  |
| **Elective cesarean section** | 427 | 4.7 |  | 17 | 2.6 | 0.56 | 0.34-0.93 |  | 26 | 6.2 | | 1.32 | | | | 0.84-2.06 | | |
| *Delivery ≤5 years of the assault* |  |  |  | 2 | 0.7 | 0.29 | 0.07-1.18 |  | 12 | 4.9 | | 0.92 | | | | 0.52-1.61 | | |
| *Delivery >5 years of the assault* |  |  |  | 15 | 4.0 | 0.91 | 0.53-1.56 |  | 14 | 8.0 | | 1.13 | | | | 0.65-1.97 | | |
| **Total^c^** | **8699** | **89.5** |  | **629** | **6.5** |  |  |  | **396** | **4.1** | |  | | | |  | | |
| **Emergency cesarean section** | 842 | 9.7 |  | 70 | 11.1 | 1.15 | 0.89-1.49 |  | 47 | 11.9 | | 1.23 | | | | 0.91-1.65 | | |
| *Delivery ≤5 years of the assault* |  |  |  | 32 | 11.7 | 1.21 | 0.84-1.75 |  | 26 | 11.1 | | 1.09 | | | | 0.75-1.58 | | |
| *Delivery >5 years of the assault* |  |  |  | 38 | 10.7 | 1.23 | 0.88-1.70 |  | 21 | 13.0 | | 1.24 | | | | 0.80-1.92 | | |

**Comparison of delivery interventions among women exposed versus non-exposed to sexual violence: By time from assault to delivery**

| **Instrumental vaginal delivery** | 698 | 8.0 | 65 | 10.3 | 1.29 | 1.00-1.66 | 28 | 7.1 | 0.88 | 0.61-1.28 |
| --- | --- | --- | --- | --- | --- | --- | --- | --- | --- | --- |
| *Delivery ≤5 years of the assault* |  |  | 33 | 12.1 | 1.28 | 0.92-1.78 | 14 | 6.0 | 0.74 | 0.43-1.27 |
| *Delivery >5 years of the assault* |  |  | 32 | 9.0 | 1.30 | 0.92-1.83 | 14 | 8.7 | 1.14 | 0.70-1.87 |
| **Emergency instrumental delivery^d^** | 1540 | 17.7 | 135 | 21.5 | 1.21 | 1.02-1.44 | 75 | 18.9 | 1.07 | 0.85-1.36 |
| *Delivery ≤5 years of the assault* |  |  | 65 | 23.8 | 1.24 | 0.99-1.56 | 40 | 17.0 | 0.94 | 0.70-1.25 |
| *Delivery >5 years of the assault* |  |  | 70 | 19.7 | 1.26 | 1.00-1.59 | 35 | 21.7 | 1.20 | 0.87-1.65 |

^a^Those who attended more than once were categorized according to age at first attendance and date of the latest assault leading to attendance to the Rape Trauma Service.

^b^Relative Risks with non-exposed women as a reference group. Data matched on age, parity and season and year of delivery. Time stratified analyses were adjusted for age, parity and year of delivery.

^c^Women who underwent elective cesarean section were excluded from all other analyses in this table.

^d^Either emergency cesarean section or instrumental vaginal delivery.
