# Supplementary material for: Obstetric Outcomes of Mothers Previously Exposed to Sexual Violence
Source: PLoS One. 2016 Mar 23;11(3):e0150726. doi: 10.1371/journal.pone.0150726 (PMC4805168; doi:10.1371/journal.pone.0150726)
Supplement: S4 Table — Labor characteristics and delivery interventions among women exposed versus non-exposed to sexual violence: Restricted to each exposed woman‘s first subsequent delivery following the exposure. (DOCX) [file pone.0150726.s004.docx]

| **S4. Supplementary table D**  **Labor characteristics and delivery interventions among women exposed versus non-exposed to sexual violence:**  **Restricted to each exposed woman‘s first subsequent delivery following the exposure** | | | | | | | | |
| --- | --- | --- | --- | --- | --- | --- | --- | --- |
| **Non-exposed women** | | | | **Exposed women,**  **first subsequent delivery** | | | | |
|  | **n** | **%** | **n** | | **%** | **RR^a^** | **95% CI** |  |
| **Total^b^** | **8699** |  | **641** | |  |  |  |  |
| **Induced labor** | 2273 | 26.1 | 196 | | 30.6 | 1.09 | 0.97-1.22 |  |
| **Labor dystocia** | 729 | 8.4 | 80 | | 12.5 | 1.10 | 0.88-1.36 |  |
| Prolonged first stage  of labor | 292 | 3.4 | 38 | | 5.9 | 1.39 | 1.00-1.93 |  |
| Prolonged second stage  of labor | 356 | 4.1 | 37 | | 5.8 | 0.98 | 0.71-1.35 |  |
| **Maternal distress during**  **labor and delivery** | 96 | 1.1 | 13 | | 2.0 | 1.37 | 0.77-2.44 |  |
| **Antepartum bleeding** | 96 | 1.1 | 16 | | 2.5 | 2.16 | 1.27-3.65 |  |
| Placental abruption | 36 | 0.4 | 6 | | 0.9 | 2.29 | 0.98-5.32 |  |
| **Emergency cesarean section** | 842 | 9.7 | 82 | | 8.9 | 1.12 | 0.90-1.39 |  |
| **Instrumental vaginal delivery** | 698 | 8.0 | 79 | | 12.3 | 1.13 | 0.91-1.41 |  |
| **Emergency instrumental delivery^c^** | 1540 | 17.7 | 161 | | 25.1 | 1.12 | 0.98-1.29 |  |
| **Total^b^** | **9126** |  | **667** | |  |  |  |  |
| **Elective cesarean section** | 427 | 4.7 | 26 | | 3.9 | 1.14 | 0.77-1.69 |  |

^a^Relative Risks with non-exposed women as a reference group. Adjusted for age, parity and year of delivery.

^b^Women who underwent elective cesarean section were excluded from all other analyses in this table.

^c^Either emergency cesarean section or vaginal instrumental delivery.
